# Synergistic Role of Plant Extracts and Essential Oils against Multidrug Resistance and Gram-Negative Bacterial Strains Producing Extended-Spectrum β-Lactamases

**DOI:** 10.3390/antibiotics11070855

**Published:** 2022-06-26

**Authors:** Manzar Alam, Nilofer Bano, Taufeeq Ahmad, Amit Baran Sharangi, Tarun Kumar Upadhyay, Yasser Alraey, Nadiyah M. Alabdallah, Mohd Ahmar Rauf, Mohd Saeed

**Affiliations:** 1 Department of Biosciences, Integral University, Lucknow 226026, India; 2 Department of Bioengineering, Integral University, Lucknow 226026, India; nilofar.160@gmail.com; 3 Faculty of Biosciences, Shri Ramswaroop Memorial University, Lucknow 225003, India; taufeeqahmadzz@gmail.com; 4 Department of Plantation Spices Medicinal and Aromatic Crops, Bidhan Chandra Krishi Viswavidyalaya, Mohanpur 741252, India; profabsbckv@gmail.com; 5 Department of Biotechnology, Parul Institute of Applied Sciences and Centre of Research for Development, Parul University, Vadodara 391760, India; 6 Department of Clinical Laboratory Sciences, Central Research Laboratory, College of Applied Medical Sciences, King Khalid University, Abha 62559, Saudi Arabia; yahamd@kku.edu.sa; 7 Department of Biology, College of Science, Imam Abdulrahman Bin Faisal University, P.O. Box 1982, Dammam 31441, Saudi Arabia; nmalabdallah@iau.edu.sa; 8 Department of Pharmaceutical Sciences, Wayne State University, Detroit, MI 48201, USA; ahmarrauf2@gmail.com; 9 Department of Biology, College of Sciences, University of Hail, Hail 4464, Saudi Arabia; mo.saeed@uoh.edu.sa

**Keywords:** plant extract and essential oil, pathogenic microbes, extended-spectrum β-lactamases, antimicrobial resistance, antioxidant, synergistic effect

## Abstract

Plants, being the significant and natural source of medication for humankind against several ailments with characteristic substances hidden on them, have been recognized for many centuries. Accessibility of various methodologies for the revelation of therapeutically characteristic items has opened new avenues to redefine plants as the best reservoirs of new structural types. The role of plant metabolites to hinder the development and movement of pathogenic microbes is cherished. Production of extended-spectrum β-lactamases is an amazing tolerance mechanism that hinders the antibacterial treatment of infections caused by Gram-negative bacteria and is a serious problem for the current antimicrobial compounds. The exploration of the invention from sources of plant metabolites gives sustenance against the concern of the development of resistant pathogens. Essential oils are volatile, natural, complex compounds described by a solid odor and are framed by aromatic plants as secondary metabolites. The bioactive properties of essential oils are commonly controlled by the characteristic compounds present in them. They have been commonly utilized for bactericidal, virucidal, fungicidal, antiparasitic, insecticidal, medicinal, and antioxidant applications. Alkaloids are plant secondary metabolites that have appeared to have strong pharmacological properties. The impact of alkaloids from *Callistemon citrinus* and *Vernonia adoensis* leaves on bacterial development and efflux pump activity was assessed on *Pseudomonas aeruginosa*. Plant-derived chemicals may have direct antibacterial activity and/or indirect antibacterial activity as antibiotic resistance modifying agents, increasing the efficiency of antibiotics when used in combination. The thorough screening of plant-derived bioactive chemicals as resistance-modifying agents, including those that can act synergistically with antibiotics, is a viable method to overcome bacterial resistance. The synergistic assessment studies with the plant extract/essential oil and the antibiotic compounds is essential with a target for achieving a redesigned model with sustainable effects which are appreciably noticeable in specific sites of the plants compared to the entirety of their individual parts.

## 1. Introduction

Antimicrobial resistance is one of the major challenges for the scientific community dealing either with medical science, in general, or with specific clinical trials with human and/or animal models. The elevated use/misuse of anti-toxins or antibiotic doses in human and veterinary clinical practices is mainly contributing to the occurrence of antimicrobial resistance [1]. Quick recognition in clinical research centers is imperative for the careful discovery of antimicrobial resistance. Expanded-spectrum β-lactamases (ESBLs) are an effective enzyme with an efficient and striking resistance mechanism that blocks the antimicrobial treatment of the diseases that occurs due to Gram-negative microscopic organisms and are a severe threat to the current antimicrobial weaponry. ESBLs are categorized into different classes as per their amino acid arrangement similarity match. Suitable pathogen management exercises and barriers are essential to keep away from the spreading and outbreaks of ESBL producing Gram-negative microscopic organisms [2]. As pathogenic microorganisms adopted various mechanisms to oppose the antimicrobial properties, the recognition of the resistant mechanism can assist with detection as well as development of novel antibacterial agents. Carbapenems are broadly considered to be the preferred medications to fix serious pathogenicity caused by ESBL-delivering Gram-negative bacteria, although available comparative clinical trials data are insufficient [2]. β-lactam anti-toxins uncover the most successive activity for microbial pathogens and persist as the significant source of resistance against β-lactam antimicrobials amongst Gram-negative bacteria across the globe. Treatment against these multidrug-resistant bacteria is of profound scientific concern. Recent ongoing studies showed a substantial change in the cases of ESBL-related diseases notified across the world [3]. Recently, there has been a hypothetical move in the subtraction of probable antibacterials. Earlier, the focuses for antibiotic invention were from microbes and at later stages the entire attention has shifted to plants. This is common because of the positive properties that plants present over those of organisms. Plant-based antimicrobials have excellent remedial worth. They additionally reduce the reactions which are regularly seen with synthetic anti-infection agents as the plant extracts are essentially composed of numerous secondary metabolites (alkaloids, steroids, flavonoids, phenol mixes, tannins, and so forth) [4]. Few plants have been demonstrated to have antibacterial properties against β-lactamase delivering multidrug-resistant Gram-negative microbes. As indicated by a report by the World Health Organization, there is an estimation that 65–80% of the world medicinal services follow the conventional strategy for drugs [5] (WHO 2016). The present review article will discuss a couple of antibiotics, plant extracts, and essential oils which have uncovered detectable effective actions against multidrug-resistant Gram-negative microorganisms that are producing β-lactamase.

Natural products are considered to be the most preferred group of structures evolved to react with a broad range of protein targets for definite action. Numerous efforts have been directed toward exploring the possible importance of herbal extracts and a few dynamic chemical formulations for their adequacy to fight against the issues of antibacterial resistance [6]. The plant extracts comprising a complex mixture of significant compounds and their secondary metabolites nearby traditional anti-infection agents produce probable combined impacts. It is well acknowledged that several illnesses with complicated pathophysiology develop an inclination for pharmaceutical mixtures [7].

Numerous favorable circumstances of using natural products as antimicrobial agents include fewer undesirable side effects, improved patient tolerance, and comparatively less cost-effective, broad approval due to their conventional advantages, sustainability, and improved biodegradability [8]. Several plant extracts from different plant parts have been broadly investigated through many experiments to understand their capacity in balancing microbial medication tolerance and these reference studies will give promising direction to forthcoming experimental studies on the inversion of antimicrobial resistance [9].

Plants produce a wide range of secondary metabolites which act as natural security to protect from the bacteria and bugs, such as coloring, aroma, or pollinator attractants. Essential oils (or unpredictable oils) are the result of the secondary metabolites derived from aromatic plants. Generally, essential oils are synthesized during steam refining or mechanical expression, whereas basic herbal extract regularly involves solvent utilization, for example, acetone, ethanol, or hexane for extraction [10]. During refining, steam condensate is isolated by gravity, giving a modest quantity of essential oil. Thus, the highly concentrated nature of essential oils may be attributed to the extraction procedure. Different essential oils have been studied to understand distinctive biological significance such as anti-inflammatory, sedative, stomach related, antimicrobial, antiviral, and cancer prevention agents [11].

Here, we discuss the synergistic role of plant extracts and essential oils against multidrug resistance in a Gram-negative bacterial strain producing extended-spectrum β-lactamases and we also confer the scientific advances that may help to overcome challenges in natural plant-based drug discovery. In the concluding section, we highlight promising future directions for natural plant-based drug discovery.

## 2. Antibacterial-Resistance Mechanism

**Figure 1:**
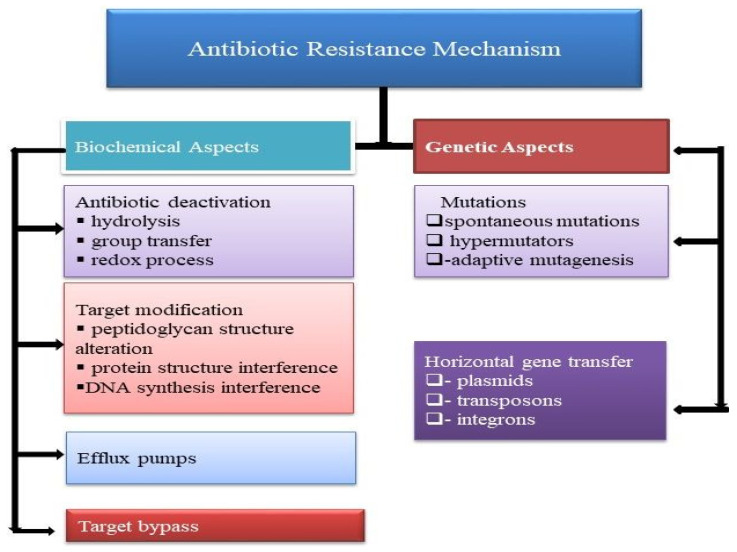
Biochemical and genetic characterization of antibiotic resistance mechanism in bacteria.

Anti-infection resistance is the decreasing inadequacy of a medication, for example, an antibacterial or an antineoplastic in restoring sickness. Whenever the antimicrobial is not intended to restrain a pathogen, the concept, at that point, is comparable with dose failure or medication resistance. Generally, the term is utilized with regard to the resistance, which is “acquired” by pathogens; it means resistance has been developed. At the point when a life form is impervious to more than one medication, it is believed to have multidrug resistance [12]. Microbial strains may have various mechanisms for resistance as shown (Figure 1).

### 2.1. Modification of Antibacterial Configuration

Numerous anti-infection agents, for example, amides and esters (having hydrolytical susceptibility), possess chemical bonds. Some known enzymes destroy the anti-infection movement by focusing on and breaking these bonds. Such enzymes can discharge often. Expanded spectrum β-lactamases (ESBLs) intervene with protection from the entire penicillin group, third era cephalosporins (for example, ceftazidime, cefotaxime, and ceftriaxone), and aztreonam, but not against cephamycins (cefoxitin and cefotetan) and carbapenems [13]. To determine the issue of anti-toxin modification by means of the enzyme β-lactamase, few methodologies have been explored. Amongst them, the most preferred and effective strategy includes a combination treatment of β-lactam anti-infection with inhibitors for β-lactamase, for example, clavulanic corrosive and tazobactam [14]. Alteration of the configuration of an existing antimicrobial is a different effective strategy to determine the β-lactamase enzyme working. More recent cephalosporins have recently been changed with the evacuation of the aminoadipoyl side chain to produce 7-aminocephalosporanic acid and to make it 100 times more successful (to make it effective) than the older age cephalosporin [15,16,17] (Table 1).

### 2.2. Modification of the Antibacterial Target Site

Alteration of the target location of anti-infection agents is one of the powerful methodologies that microbes can utilize to endure antimicrobial treatment. Such a strategy centers around modification regarding the structure of the anti-toxin target and this can happen in two unique manners: through hereditary transformation and additionally through post-transcriptional and translational changes. β-lactam anti-toxins, for example, penicillin, primarily target and inactivate a transpeptidase penicillin-binding protein (PBP) during peptidoglycan cell wall cross-linkage by acylating the enzyme active site. Mutation can convert the PBP gene into a different form of a useful protein that blocks binding of the β-lactam anti-toxins, such as penicillin [18,19]. Examples of antibiotic-resistant strains/microorganisms with this ability are *Clostridium difficile*, *Enterococcus faecium,* and *Streptococcus pneumoniae* [20,21].

### 2.3. Antibacterial Efflux Pump and Reduced Permeability

Efflux pumps are the proteins, considered to be active transporters that serve to oust or to avoid the passage of noxious compounds. For this situation, anti-infection agents are introduced into the microbial cytoplasm, with the assistance of energy either from ATP (adenosine triphosphate) or from an electrochemical potential [19]. Microscopic organisms, for example, *Pseudomonas aeruginosa* and *Escherichia coli*, have been seen as widely resistant to fluoroquinolones and ciprofloxacin because of the proteins’ overexpression associated with the efflux pump mechanism controlled by the H^+^ ion gradient [22].

Certain microscopic organisms, for example, *P. aeruginosa* and methicillin-resistant *Staphylococcus aureus* (MRSA), can express non-specific multidrug resistant pumps, rather than having an efflux pump system. Such multidrug resistant pumps efflux a range of anti-toxins including the β-lactam anti-infection agents [23,24].

Microscopic organisms’ intake and transport amino acids, small polar particles, and supplements through ‘porins’ (water-filled channel proteins). In any case, the porin channels which are hydrophilic in nature additionally permit interference from anti-infection agents, for example, hydrophilic cephalosporins and penicillin N. To avoid such occurrence, microscopic organisms can decrease and additionally silence stop the interaction of such porin channels and can alter the structure of the porin protein channels by decreasing permeability of such anti-toxins [24,25].

### 2.4. Antibacterial Deactivation by Group Transfer 

Transferases are mainly considered to be the different group of resistant enzymes that inactivate anti-infection agents (chloramphenicol, aminoglycosides, streptogramin, rifampicin, or macrolides) by replacement of chemical moieties (adenylyl, phosphoryl, or acetyl groups are added to the periphery of the antimicrobial molecules). The altered anti-toxins are influenced in their binding with specific targets of chemical methodologies which include N-acetylation and O-acetylation [26,27], O-phosphorylation [28,29], O-nucleotidylation, O-ribosylation [30], O-glycosylation, and thiol transfer. All such covalent modification procedures require a co-substrate molecule for their action (ATP, NAD+, UDP-glucose, glutathione, or acetyl-CoA) and, thus, these procedures are specifically limited within the cytoplasm.

### 2.5. Rehabilitation of Cell Wall

Under a typical physiological environment, the bacterial cell wall is continuously being restored with the previous peptidoglycan-rich cell wall being separated and supplanted or replaced by new molecules. Anti-toxins from the glycopeptide group are considered to be the main target during the development of the bacterial cell wall. β-lactam antimicrobials such as cephalosporins and penicillin interfere with proteins which are essential for peptidoglycan layer synthesis. Glycopeptides (teicoplanin, vancomycin, oritavancin) focus on the cell wall of the bacteria by adhering to the D-alanyl-D-alanine ends present in the peptidoglycan sequence and block the cross-linking steps in this manner. Telavancin, a novel lipoglycopeptide with strong bactericidal action, represses biosynthesis of peptidoglycan through targeting transglycosylation specifically [31]. This blocks the bacterial cells from replacing their old peptidoglycan wall, which, due to treatment, can be frequently broken down in the long run through lysing and death of the cell.

Vancomycin-resistant *Enterococci* sp. generates an altered peptidoglycan precursor, which is produced from peptidyl-D-Ala-D-Ala to peptidyl-D-Ala-D-Lac or peptidyl-D-Ala-D-Ser and prevents antimicrobial glycopeptide binding [32]. Accomplishing such modification requires the acquisition of genes as well as natural hereditary change. Genes, for example, the vanA group, encode two specific enzymes: VanH dehydrogenase that changes pyruvate into D-Lac and VanA ligase enzyme that encourages the esterification of D-Ala and D-Lac to form peptidyl-D-Ala-D-Lac [33,34]. Moreover, mutation of Dd1 ligase at the individual amino acid level, which is required for the ligation of two D-Ala, allows them to join with D-Ala to D-Lac, accordingly, giving resistance to glycopeptide anti-toxins. This occurrence had likewise recently been seen in a vancomycin-resistant *E. coli* strain [35].

## 3. ESBL Definition and Categorization

**Figure 2:**
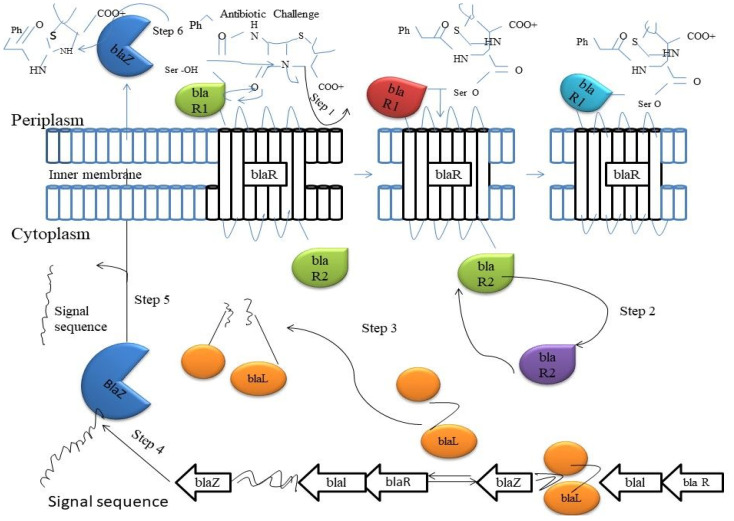
The upregulation of the Bla enzymes occurs when the two-component signaling system comes into contact with β-lactam antibiotics. A molecule of β-lactam antibiotic binds to active-site serine of the BlaR1 enzyme on the outside of outer membrane (Step 1). A conformational change occurs that releases the BlaR2 on the inside of the cellular envelop (Step 2). BlaR2 cleaves Blar1 of the DNA to allow for the transcription of downstream resistance genes (Step 3). The β-Lactamase BlaZ is produced from downstream genes (Step 4). BlaZ is transported out the cell, and the signal sequence is cleaved off the protein (Step 5). The BlaZ β-lactamase starts hydrolyzing incoming antibiotics (Step 6).

There is no agreement about the exact meaning of ESBLs. ESBLs are a cluster of enzymes which separate the antimicrobial association with cephalosporin and penicillin groups and make them non-functional. ESBL has commonly been characterized as transmittable β-lactamases which can be repressed by clavulanic acid, tazobactam, or sulbactam, and are decided by genes that can be interchange among microscopic organisms. Presently, CTX-M is the most regular hereditary variation of ESBL [36]. Generally, β-lactamases are grouped by two general plans: the Ambler molecules order and the Bush–Jacoby–Medeiros useful categorization [37,38]. The Ambler system categorizes β-lactamases into four groups as per the protein similarity of enzymes. Classes A, C, and D of β-lactamases are serine β-lactamase and enzymes from class B are metallo-β-lactamases. The Bush–Jacoby–Medeiros useful plan depends on functional properties of enzymes, for example, the substrate and inhibitor profiles (Table 1 and Figure 2).

### 3.1. Type SHV

The SHV group of β-lactamases emerged from *Klebsiella* spp. The ancestor for SHV division of enzymes, SHV-1, is commonly found in *K. pneumoniae*. In numerous strains of *K. pneumoniae*, the gene which encodes SHV-1, or its evident ancestor, LEN-1, resides within the microbial chromosome as well. It might be that the gene encodes for SHV-1 β-lactamase advanced like a chromosomal gene in *Klebsiella* and then consolidated into a plasmid which has been extended to other enteric bacterial spp. SHV-1 presents protection from an expansive range of penicillins, for example, tigecycline, piperacillin, and ampicillin; however, not for oxyimino-substituted cephalosporins [39]. The SHV-1 β-lactamase is responsible for almost 20% of the plasmid-mediated resistance against ampicillin in *K. pneumoniae* spp. [40]. 

### 3.2. Type TEM

In 1965, TEM-1 was initially detailed from *E. coli* isolates. TEM-1 has substrate and hindrance description like that of SHV-1 [41]. It has the capability to hydrolyze penicillins and cephalosporins (first-generation), however, cannot assault the oxyimino cephalosporin. The variant TEM-3, first TEM generation, emerges from the enlarged movement against extended-spectrum cephalosporins [2]. TEM-2, the primary subordinate of TEM-1, had a solitary amino acid replacement from the first β-lactamase. This leads to a transfer in the isoelectric point from a pI of 5.4–5.6, yet it did not modify the substrate profile. In 1989, TEM-3 was initially detailed as the first TEM-type β-lactamase that exhibited the ESBL phenotype [2].TEM-3 might not have been the first TEM-type ESBL. *Klebsiella oxytoca*, holding a plasmid handover gene which encodes ceftazidime resistance, was reported for the first time in Liverpool, England, during 1982 [42]. The β-lactamase dependable was what is presently called TEM-12. The isolates originated from a neonatal unit that had been suffering from the occurrence of *K. oxytoca* creating TEM-1 [42]. This occurrence is a genuine case of the development of ESBLs as an effect on the specific and selective force induced and instigated by extended-spectrum cephalosporins 

### 3.3. Type CTX

Another group of β-lactamases which specially hydrolyzes cefotaxime has emerged. It is reported mainly in isolates of *E. coli*, *Typhimurium*, and *Salmonella entericaserovar*, and few other species from Enterobacteriaceae [2,43]. These types are not firmly identified with SHV β-lactamases or TEM [44]. Along with the quick cefotaxime hydrolysis, an extra special advantage of these enzymes is that they are effectively repressed by the β-lactamase inhibitor tazobactam rather than by clavulanate and sulbactam [2]. CTX-M β-lactamase originates entirely in the functional group 2 [45] and is considered to begin from chromosomal ESBL genes exhibited in *Kluyvera* spp. (originated as an opportunistic pathogen of the Enterobacteriaceae). In the late 1980s, the first CTX-M proteins were found and, these days, an excess of 100 variations have been sequenced [13]. Based on their amino acid sequences, they can be separated into five classes (CTX-M bunches 1, 2, 8, 9, and 25) [13]. The main source from where CTX-M enzymes originate is not the same as that of TEM and SHV ESBLs. TEM-ESBLs and SHV-ESBLs were created by replacement of amino acids from their parent enzymes, CTX-M. ESBLs were procured by the horizontal gene transfer from different microscopic organisms utilizing hereditary mechanical apparatuses, for example, transposon or conjugative plasmid. The gene sequences which encode CTX-M enzymes exhibit high comparability to those of β-lactamases of *Kluyvera* spp. Moreover, genetic sequences near the CTX-M genes of *Enterobacteriaceae* are additionally like those nearby the β-lactamase qualities on the chromosomes of *Kluyvera* spp. [46,47].

### 3.4. Type OXA

The β-lactamases OXA-type are named due to their oxacillin-hydrolyzing capacities. Such β-lactamases are categorized by hydrolysis rates for oxacillin and cloxacillin superior to 50% as that for benzyl penicillin [48]. It mostly happens in *P. aeruginosa* [49] yet has been distinguished in other numerous Gram-negative microorganisms. Actually, the most commonly recognized OXA-type β-lactamase, OXA-1, has originated in 1–10% of *E. coli* isolates [2]. The ESBLs OXA-type was initially found in *P. aeruginosa* isolates from a solitary emergency clinic in Ankara, Turkey. A novel derivative of OXA-10 (numbered OXA-28) originated in a *P. aeruginosa* isolate in France [50]. A new ESBL (OXA-18) and an all-extended-spectrum derivative for the narrow spectrum OXA-13 β-lactamase (numbered OXA-19) have additionally been found in *P. aeruginosa* isolates in France [51]. The ESBL OXA-type β-lactamases was developed from relative enzymes with smaller spectra that have multiple similarities with the advancement of SHV-and TEM-type ESBLs. Regrettably, there is not much epidemiologic information on the geological extent of ESBLs OXA-type [51].

### 3.5. PER Type

The PER-type ESBLs have an approximately 25–27% homology match with recognized SHV-type and TEM ESBLs [52]. PER-1 β-lactamase can effectively hydrolyze the cephalosporins and penicillins and is vulnerable to clavulanic acid resistance. PER-1 was first distinguished in *P. aeruginosa* [53] and later in *Typhimurium, Acinetobacter*, and *S. enterica serovar* isolates too [54].

### 3.6. Type GES

GES-1 type was at first reported in isolates from *K. pneumoniae* from a newborn patient who just moved to France from French Guiana [50]. It has hydrolytic action among expanded spectrum cephalosporins and penicillins; however, not against carbapenems or cephamycins and is restrained by β-lactamase inhibitors. Such enzymatic qualities take after those of different classes. Consequently, GES-1 type was characterized as an individual from ESBLs.

### 3.7. VEB-1, BES-1 and Other ESBL Types

Several abnormal enzymes having ESBL (for example CME-1, BES-1, PER, SFO-1, VE-B-1, and GES-1) have also been reported [55]. Such original proteins are established rarely, and information on these enzymes is examined elsewhere [56].

## 4. Detection

Curing of contamination due to ESBL-producing microorganisms with aztreonam or extended-spectrum cephalosporins possibly may bring about treatment collapse in any event when the causative microbe emerges as vulnerable to such antibacterial agents using regular vulnerability testing [48]. Furthermore, patients exposed to or contaminated with ESBL-producing microorganisms ought to be put under contact safeguards to stay away from emergency clinics [57]. The above-mentioned advantages gather the findings of ESBL-producing microorganisms in clinical research facilities. The European Committee on Antimicrobial Susceptibility Testing (EUCAST) has made a correction to cephalosporin breakpoints. Further works are in progress by the Clinical and Laboratory Standards Institute (CLSI) on the enhanced forecast of the clinical result based on MIC values [58]. It is, so far, conflict-ridden whether this update may permit clinical research centers to be abstaining from ESBL identification [58].

### 4.1. Phenotypic Identification

For ESBL recognition in Enterobacteriaceae, specifically for *E. coli, Proteus* spp., and *Klebsiella* spp., rules have been distributed by the US Clinical and Laboratory Standards Institute (CLSI) and the UK Health Protection Agency (HPA) [59,60]. HPA rules additionally incorporate different species, for example, *Salmonella* spp. This depends on the rule that many ESBLs hydrolyze third-generation cephalosporins in spite of the fact that they are hindered by clavulanate, which then suggests the onset of screening with a dose of 8 mg/L (CLSI) or another dose of 1 mg/L (HPA) of cefpodoxime, 1 mg/L each of ceftazidime, cefotaxime, aztreonam, or ceftriaxone, followed by corroborative tests (counting E-test ESBL strips) with both ceftazidime and cefotaxime combined with clavulanate at a convergence at 4.l g/mL. Computerized frameworks with utilization-comparable recognition standards have ended up being well known in clinical research centers, particularly those in certain European nations and North America [61]. In the event that clinical research facilities hold fast to the distributed rules for identifying ESBLs, the HPA and CLSI distributed techniques show increased affectability of upto 94% and particularity at 98% for recognizing ESBLs in *Klebsiella*, *E. coli* spp., and *Proteus* spp. [62].

### 4.2. Genotypic Identification

The assurance of whether or not a selected ESBL gift in clinical isolates is known with SHV and TEM enzymes could be a confounded procedure since rational transformations regarding the active sites of the SHV and TEM groupings have prompted amino acid transformation that expanded the range of movement of the parent enzymes, for example, in TEM1, TEM2, and SHV1 [55]. The typically used molecular techniques include the PCR amplification of blaSHV and blaTEM qualities with oligonucleotide primers, followed by sequencing. To discriminate between the non-ESBL parent enzymes (for example TEM1, TEM2, or SHV1) and numerous variations of TEM or SHV ESBLs (for example, TEM3, SHV2, and so on), sequencing is one of the most important methods [55]. PCR amplification of CTX-M-explicit items without sequencing, in an isolate which delivers ESBL, for the most part, provides adequate proof that a blaCTX-M sequence is responsible for this phenotype. This is often dissimilar to SHV and TEM varieties of ESBLs. It is described that different molecular methodologies are used for rapid screening of ESBL-positive microorganisms for the occurrence of assorted blaCTX-M genes. It incorporated a PCR assay where four sets of primers were used to accentuate clusters expressing CTX-M β-lactamase genes [63], the amplification of a general deoxyribonucleic acid piece expressed for the bulk of the varied cluster of CTX-M β-lactamases duplex PCR [63,64], multiplex PCR [65], a period of pyrosequencing [56], time PCR [66], and invert line hybridizing [67]. The molecular assays, no doubt, will presumably have a basic influence within the center system for the screening, tracking, and observation of unfolding of a vast variety of microorganisms that produce CTX-M enzymes from the emergency clinic and community. 

## 5. Role of Plant Secondary Metabolites as Antimicrobial Agents

Plants produce a large assortment of secondary compounds as natural protection against microbes. A portion of these compounds are equally poisonous for mammals; however, others may not be harmful. Without a doubt, a significant number of these compounds have been utilized as entire plants or herbal extracts for food or clinical applications (Table 2). Generally, plants have limitless capability to synthesize aromatic substances; the greater part in them is phenols or their oxygen-subbed subordinates [68]. Almost 12,000 secondary metabolites have been isolated, of which 10% of the aggregate is under evaluation [69]. Much of the time, these substances fill in as plant protection machinery against predation by herbivores, insects, and microorganisms. A few, for example, terpenoids, give their odor to plants; others (tannins and quinones) are responsible for the herbal pigment. Plants produce a broad range of compounds that may not be significant for primary metabolism yet represent a versatile competence of a plant regarding antagonistic biotic and abiotic natural conditions [70]. Such organic compounds are biologically dynamic and represent ‘secondary metabolites’, which are the product of secondary plant metabolism and occur as intermediate or final results [70]. During development, structures of secondary metabolites have been advanced, due to which they can act as defense components by intervening with molecular targets of the microbes [71]. Furthermore, few secondary metabolites act against oxidative or UV stress or also influence cell signaling [72] (Table 3).

### 5.1. Antimicrobial Activity of Plants

Antibiotic resistance is becoming more common among bacterial pathogens, necessitating the use of medicinal plants as an alternative therapy for limiting the spread of resistant pathogenic organisms. Plant extracts and phytochemicals, both of which are known to have antibacterial characteristics, can be very useful in therapeutic treatments. A number of studies have been undertaken in various nations in recent years to demonstrate such efficacy [96]. Many plants have been employed for their antibacterial properties, which are related to chemicals produced in the plant’s secondary metabolism. These items are recognized by their active ingredients, such as phenolic compounds found in essential oils [97] as well as tannins [98]. A number of researchers from all around the world, particularly in Latin America, have looked into the antibacterial characteristics of plants [96]. Antibacterial activity was dependent on the bacteria species, plant species, and extract type (Table 4).

### 5.2. Essential Oils

**Figure 3:**
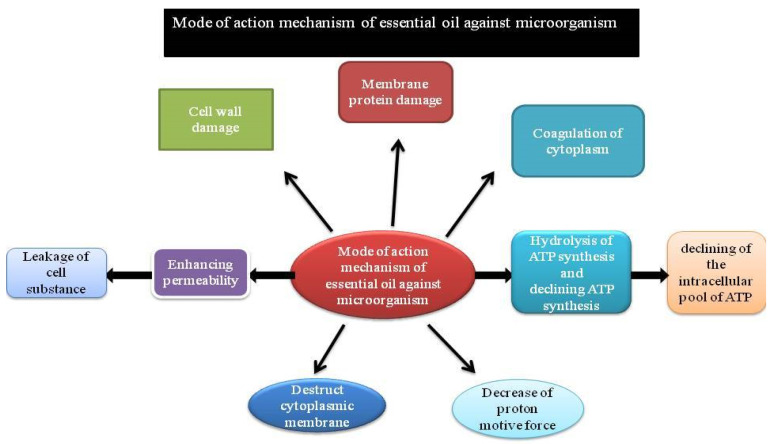
Schematic representation of the mode of action of essential oils.

Human beings have used essential oils for a long time, which are organic-solvent extracts from plants or steam-volatile compounds. They can be used due to the nice odor of their essence, their flavor, and their antibacterial and/or additive properties. They are used in various plants for their protection responsibility towards microorganisms, insects, or microflora attack. Essential oils are regarded as natural substitutes to chemical biocides and antibiotics along with some other applications. Recently, the helpful property of essential oils has been indisputable among infectious microorganisms. Oils extracted from *Cinnamomum osmophloeum* show medicinal drug activity towards *Staphylococcus aureus*, *E. coli*, *Salmonella* spp.(including methicillin-resistant *S. aureus,* which is the clinically problematic strain), *Enterococcus faecalis*, and *Vibrio parahemolyticus*; cinnamaldehyde is the major medicinal drug constituent of the combination [100]. Oregano oil inhibits *E. coli* O157:H7 peppermint oil [101,102] and essential oils from other herbs (Marino et al. 2001). *H. pylori* are susceptible to spearmint oil [101]. These essential oils are effective among a broad range of oral microorganisms [103], and they can be used broadly in antibacterial mouthwashes. Some years ago, essential oils were investigated in rumen microorganisms as they are involved in reduction of deliciousness in a few plant varieties [104]. Commonly, essential oils demonstrate a lot of efficiency towards Gram-positive organisms compared to Gram-negative microorganisms [105,106]. It has been hypothesized that the occurrence of lipopolysaccharides surrounding the microorganism, which constitutes the peptidoglycan layer, has inhibited the mechanism of diffusion of compounds (which are hydrophobic in nature) into the cytoplasm [107]. However, it is not concluded in all studies on essential oils that Gram-positives are an variety of vulnerable organisms [108]. Fascinatingly, in a study from van Vuuren et al. (2009) [109], a mixture of the essential oil of *Rosmarinus officinalis* with addition of ciprofloxacin towards Gram-positive microorganisms gave an antagonistic property, whereas *Rosmarinus officinalis*/antibiotic towards Gram-negative microorganisms showed an excellent synergistic profile [109]. Since only insufficient sample sets have been considered, variety of the entire test would greatly enhance the properties of essential oils as resistance modifiers of antimicrobials in infectious studies (Figure 3).

### 5.3. Alkaloids

Generally, alkaloids are heterocyclic nitrogen compounds. The major clinically supportive example of an alkaloid was morphine, separated from the opium poppy (*Papaver somniferum*) in 1805 [110]. The name morphine has been derived from the Greek God who is the “God of dreams”. Both heroin and codeine are derivatives of morphine. Diterpenoid alkaloids are normally separated from the plants of Ranunculaceae, uniquely possessing antibacterial properties [111]. Solamargine, a glycoalkaloid from berries of *Solanum khasianum* and other alkaloids could also be helpful against AIDS [112] for enteric infection related to HIV [113]. Whereas alkaloids are found to possess a germicidal property towards *Entamoeba* species and flagellated protozoans [114], the foremost medicinal drug result is because of their effecton transportation time inside the intestine. Berberine is considered to be the significant agent of alkaloid clusters. It is potentially efficient among plasmodia [115] and trypanosomes [116]. The action mechanism of extremely aromatic planar quaternary alkaloids such as harmone and berberine [117] is recognized for its capability to insinuate with DNA [118].

### 5.4. Phenolics

The substituted phenolic ring is mainly present in some of the bioactive phytochemicals. Caffeic and cinnamic acids are common representatives of a large cluster of phenyl propane-derived compounds with the highest oxidation number. Both the herbs thyme and tarragon contain caffeic acid, which is effective against viruses [119] and also against fungi and microorganisms [86]. Pyrogallol and catechol are hydroxylated phenols, which are deadly to microorganisms. Pyragallol has three -OH groups while catechol possesses two. The sites and variety of -OH groups on the phenol are considered to be associated with its relative toxicity against microorganisms, with proof that elevated hydroxylation results in amplified toxicity [119]. Additionally, some authors have found that a lot of extremely oxidized phenols are related to a great deal of defense mechanisms [120]. The procedure considered to be accountable for phenolic toxicity against microbes is when there is enzyme hindrance from the oxidized moieties, possibly during the reaction with sulfhydryl (-SH) groups or through a lot of indefinite connections with the proteins [121]. Phenolic molecules containing a C3 side chain at a lesser degree of oxidization and with an ingredient are categorized and cited as essential oils or as antibacterials. Eugenol, a well-known chemical compound in clove oil, is considered to be antiseptic among both fungi and microorganisms [86].

## 6. Plant Extracts against the ESBL-Producing Multidrug Resistant (MDR) Gram-Negative Bacteria

The number of ESBL-delivering MDR microbes, for example, *Klebsiella* spp. and *E. coli*, is rising because of the determination pressure applied by the abuse of antimicrobials [122]. Pathogens caused by ESBL-producing *E.coli* and *Klebsiella* spp. have been increasing in recurrence as well as in complexity [123]. The utilization of plant extracts for therapeutic use became recognized when individuals understood that the limited life span of antitoxins, over remedy and abuse of traditional anti-infection agents is causing antibacterial resistance [124]. Traditionally utilized medicinal plants produce different types of compounds with therapeutic importance [125]. Choudhury et al. (2013) [126] described that the *Allium sativum* (bulb), *Allamanda cathartica* (leaf), *Citrus limon* (organic product), *Tamarindus indica* (natural product), *Averrhoa carambola* (organic product), *Prunus domestica* (fruit), *Terminalia arjuna* (leaf), and *Piper betle* (leaf) showed likely antimicrobial properties against the clinical bacterial isolates. In *Tamarindus indica*, the most extreme antimicrobial action was observed to be 80% in the event of *Pseudomonas aeruginosa*, *Proteus mirabilis*, and *Klebsiella pneumoniae* isolates, then *Averrhoa carambola* showed the greatest action against *Escherichia coli* isolates. Tetrahydrosecamine separated from *R. stricta* shows a wide range of antimicrobial action, and Strictanol, another active constituent was seen as most active towards *Pseudomonas aeruginosa* and *E. coli* [127]. The rough ethanolic extracts of *R. stricta* natural products have lipoxygenase, acetylcholinesterase, and antibacterial activities [128]. The methanolic extracts of *R. stricta* roots and chloroform show antifungal and antimicrobial activity towards *E. coli, Bacillus subtilis*, *Aspergillus terreus*, *P. aeruginosa*, *S. aureus*, *Candida albicans*, and *Aspergillus flavus* [128].

## 7. Mode of Action of Plant-Derived Drugs

Generally, six attainable modes of antimicrobial action have been very well known [5] which includes disintegration of cytoplasmic membrane [129], disorder of the outer membrane of Gram-negative microorganism with the discharge of lipopolysaccharides [130], interaction with membrane proteins, i.e., ATPases and other enzymes [131], deterioration of the proton driving force with leakage of ions [132], natural action of the cell constituents [133], and inhibition of enzyme synthesis [134]. The results of essential oils along with their active bio-constituents principally disrupt the microorganism cell membranes followed by the discharge of membrane parts [135]. It has been reported that the constituents from lemongrass oil additionally suppressed the formation of biofilm, killed previously formed biofilms, and have many targets on the microorganism cell [135]. The monoterpenes from essential oils which are lipophilic in nature effectively act and have an effect on the lipid bilayer structure. Few examples include myrtle volatile oil that predominantly affects the structure of the membrane and cell wall resulting in the discharge of intracellular components and disruption of membrane performances such as enzyme activity, nutrient absorption, or electron transfer [136]. Lipid membrane absorbs P-cymene and carvacrol and affects the composition of membrane lipids. Antimicrobial activity of terpenes such as thymol additionally disrupts the lipid bilayer [137]. Alteration of physicochemical surface properties of uropathogenic *E.coli* by adherence to uroepithelial cells has also been studied in cranberry [138] (Table 5).

## 8. Plant-Oriented Drug Resistance

The resistance against plant medication in numerous clinical/non-clinical isolates of pathogens has been evidenced more recently from animal clinical isolates. However, this sensitivity or resistance is comparative and varied results were observed with different drug concentrations used [148]. In studies on resistance against LGO and different herbal medication, variable levels of MIC were shown [128] depending upon microbial species tested or inside similar species among completely variable strains, which suggests that microorganism have a mechanism to beat the germicidal concentration of plant medication conjointly. The capability of microorganisms to generate resistance against plant medication is still not properly studied. It is usually stated that microorganisms cannot develop resistance to plant medicines [149]. However, new reports recommended that microorganisms will overcome germicidal or biological activities of plant medication. Several herbal medications reported containing higher antimicrobial activities either individually or in combination with antibiotics. However, reports on non-functional herbal medication on specific strains cannot be neglected [128]. Several clinical and non-clinical microbial isolates were sensitive to herbal medication such as *S. aromaticum*, *T. arjuna*, and *A. nilotica*. However, studies reported that a number of isolates, such as *E. coli, P. aeruginosa*, *K. pneumoniae*, *C. albicans*, and *E. coli* from nosocomial infections and *C. albicans*, *K. Pneumonia*, and *E. coli* isolated from the community were resistant to herbal medication. Singh et al. (2011) [148] reported that many microorganism strains derived from completely different clinical complications from post-mortem cases and in animals were resistant to lemongrass oil. High resistance was observed in microorganism strains from gecko origin towards antimicrobials of herbal origin. Moreover, it was found that plant drug resistance varied between strains of microorganisms and plant medication can be specific and will not have a broad-spectrum to large microorganism populations [149]. Important use of herbal medicines is to process the multidrug resistance (MDR) pathogens. However, some MDR or drug-resistant strains have conjointly been isolated from plant material. Brown and Jiang isolated tetracycline-and ceftriaxone-resistant microorganisms from garlic soil samples at 3.0 × 10^2^ CFU/g and 1.1 × 10^2^ CFU/g, respectively [150]. Similarly, microbes such as tetracycline-resistant microorganisms were present in organic samples of onion powder. Presence of drug-resistant microorganisms within the plant products may also become an origin of antibiotic resistance [151] (Table 5).

## 9. Reversal of Antibacterial Resistance through Synergism

Many scientists have investigated and assessed the significance of the synergistic effect of plant-derived compounds and traditional antibiotics [152]. The synergistic interaction between two agents, in which one agent enhances the effect of the other and together act more efficiently than as individual agents, has prompted many scientists to investigate and assess the significance of synergistic action of plant-derived compounds and traditional antibiotics. Plant extracts are widely known for their antibacterial qualities, as well as their capacity to improve the action of an antibiotic when used in conjunction with it. This ability of plant active compounds manifests itself in the modification or inhibition of resistance mechanisms, resulting in the bacterium being susceptible to antibiotics or the antibiotic acting at lower doses. On the one hand, this strategy reduces the effective dose of antibiotics, but it also reduces the negative effects of antibiotics as medicine. Numerous in vitro studies have shown that plant extracts and antibiotics have synergistic effects, resulting in a considerable reduction in antibiotics’ minimum inhibitory concentration (MIC). Scientists have experimented with a variety of plant extracts in combination with various antibiotics. Antibiotics from the group of inhibitors of cell wall and protein production were the most common [153]. The ethanol extract of *Punica granatum* rind showed excellent synergic effect with ciprofloxacin, resulting in a 34-fold drop in MIC and re-sensitization of *Klebsiella pneumoniae* resistant strains [153]. The antibacterial and modulatory activity of ethanol extracts from *Azadirachta indica* leaves and bark in conjunction with aminoglycosides and carbapenems was investigated against *Staphylococcus aureus*, *Escherichia coli*, and *Pseudomonas aeruginosa*. The combination of ethanol bark extract and amikacin has a synergistic effect against the *P. aeruginosa PA* 24 strain. Ethanol bark extract plus imipenem, amikacin, or gentamicin had a synergistic effect against *E. coli* bacteria, while imipenem had a synergistic effect against *S. aureus* strains [154].

In response to abuse of β-lactam anti-infection agents, numerous microbial strains have been developed to synthesize β-lactamase enzymes that break the β-lactam ring structure of such antimicrobials, making them inactive. Clavulanic acid is a weak β-lactam, having insignificant innate antibacterial action despite sharing the same β-lactam ring with other β-lactam antimicrobial agents. Recently, several studies analyzing synergistic communication between herbal extracts and resistance-prone antimicrobial agents have essentially expanded. Antimicrobial herbal products in combination with drugs have become a research need because of a few components, including commercial advantage over traditional techniques for drug discoveries. In contrast with developing other drugs that require long periods of extensive testing, a point of combination treatment is to re-establish a current drug to the state of significantly reduced resistance. Re-establishing action to conventional anti-infection agents utilizing a combination would empower the drug to arrive at clinical utilization considerably more quickly and decrease the advancement cost as the active bio-component of the combination has just been assessed through a broad clinical trial. In this manner, the requirements for testing are less precise. Further, benefits of synergistic interactions include reduced side effects, expanded effectiveness, increased efficiency, bioavailability, increased stability, and the requirement for minimum doses as compared to synthetic replacement [155], the grouping of β-lactams with kaempferol or quercetin from different herbs, vegetables, fruits, and seeds [156], or with a-mangosteen separated from fruit mangosteen [157], or generously increasing the competence of the treatment in β-lactam-resistant microbial strains. Almost certainly, the mangosteen isolated parts of such combinations may repress the bacterial β-lactamase enzymes, in this way reactivating the antimicrobial. Indeed, even herb-derived combinations themselves have been observed to have antimicrobial properties, for example, berberine enhances *Psuedomonas aeruginosa* amino-glycoside resistance [158]. Hence, the capacity of herbal compounds to “repurpose” traditional antimicrobials for treatment of pathogenic diseases may remarkably affect all-inclusive wellbeing as far as combating resistant pathogenic microorganisms. The synergistic evaluation studies examine combinations of at least two drugs with expectations of accomplishing an upgraded general impact which is considerably more prominent than the sum of their individual parts [159]. In recent times, combination treatment has increased widespread acceptance and recognition, particularly in the area of pathogenic infection. Plant extracts/antimicrobial combinations not only add to and upgrade the general antimicrobial impact, yet they can equally perform as resistance-modulating/modifying specialists. Few rough plant extracts harm the cytoplasmic layer of resistant microbes which causes damage to intracellular components. The herbal extracts, combined with oxacillin activity, possibly caused considerable cell membrane perturbation. Combinational treatments may improve the movement of powerless antimicrobials against microorganisms. One drug may overwhelm or kill the mechanism of microbial resistance, repurposing the antimicrobials through expanding their competence. Maybe the most popular case of antimicrobial synergy is the grouping of β-lactam antitoxins with clavulanic acid which is a fungal-derived inhibitor of β-lactamase enzymes [160]. Ciprofloxacin synergistically interacts with *Berberis aetnensis* C. Presl a Sicilian plant [161]. Chloroform extract derived from *Berberis aetnensis* leaves remarkably decreases the minimum inhibitory concentration (MIC) of ciprofloxacin towards *P. Aeruginosa* and *E. coli*. A combination of anti-infection agents and extracts from jambolan, clove, thyme, and pomegranate showed critical synergistic association towards a multidrug resistant *P. aeruginosa* strain [162]. Likewise, the upgraded antibacterial progress was seen in a combination with clove–ampicillin and clove–antibiotic medication against *Proteus* spp. and *K. pneumoniae,* respectively [163]. Such synergistic examinations mentioned herewith, do not provide a guarantee in the fight against MDR infections, yet may likewise also prove that anti-infection agents remain ineffective when used alone. A methanolic *Petalostigma* spp. isolate associated synergistically with antibiotics chloramphenicol, erythromycin, and penicillin, to repress the development of *Proteus* spp. The strain *P. mirabilis* was especially resistant against erythromycin and chloramphenicol, and with just a decreased susceptibility against penicillin. Such anti-infection agents are helpless against resistance because of the efflux pump [163]. Current advancements in the field of science and innovation reveal the search for new antimicrobial therapy which is made progressively possible. Researchers have shifted more concentration to discovering new medications to find plant-derived compounds which can resuscitate the viability of active anti-infection agents during the methods of synergism with utilization of decreased concentrations of the anti-toxin [164]. A report showed the importance of essential oil from *Helichrysum italicum*, the curry plant which works synergistically and significantly against chloramphenicol. It can be effective in combination and remarkably minimizes the prescribed amount of antimicrobial required to obliterate *E. aerogenes*, a chloramphenicol-resistant strain. At the point when treatment of *E. aerogenes* was completed with chloramphenicol alone, then its efflux pump gets overexpressed. However, on application in combination, the researchers observed activity of essential oil as an efflux pump inhibitor, which blocks the evacuation of the chloramphenicol anti-infection empowering it to disseminate over the microbial cell wall and cell membrane. Likewise, the essential oils derived from *Cinnamomum verum*, lavender, *Lavandula angustifolia*, and cinnamon bark, appeared to display synergistic associations with piperacillin when it was used against *E.coli*, a multi-antibiotic-resistant strain [165]. Such synergism emerged from various mixes inside the essential oil that can be helpful to infuse the external membrane, and to additionally hinder quorum sensing capacity of the *E. coli* multi-antibiotic-resistant strain [166]. A larger part of the alkaloid mixes, which was determined and considered, proposed that alkaloids had the widespread capacity to hinder the efflux mechanism of antibiotic-resistant microbes. For example, lysergol responds synergistically to a quinolone, nalidixic acid, antimicrobial during inhibition of the efflux pump when it was used against *E. coli* multi-antibiotic-resistant strain [167]. Phenolic compounds, for example, epicatechin gallate and baicalin appeared to show synergism in association with anti-infection agent β-lactam when utilized among resistant isolates of Gram-positive and Gram-negative microorganisms, such as MRSA and *E.coli* [168]. Moreover, synergistic associations have been found among phenolic fractions, for example, p-coumaric acid, taxifolin, caffeic acid, and sinapic acid in combination with anti-infection agents, for example, erythromycin and ciprofloxacin when utilized towards MDR *Campylobacter jejuni*. The method of activity of specified phenolic compounds includes the restraint of the efflux mechanism just as extra penetrability of the cell membrane has been proved recently [169] (Table 6).

## 10. Future Prospects

Across the history of humans, plants have always been utilized as a therapy for the handling of the infections, before the invention of antibiotics. As an example, colchicine organic compound isolated from *Colchicum autumnale* plant is utilized in therapy towards cancer. Whereas ajmaline, a glycoside inhibitor of Na/K ATPase, separated from *Rauwolfia serpentina* plant, can be utilized against cardiac arrhythmias [176]. Regrettably, the present inventory of industrially accessible antibiotics is largely from bacteria or fungi; the only industrial antibiotics of herbal origin, artemisinin from *Artemisia annua* plant and quinine from *Cinchona officinalis* plant that were explored or proved against diseases caused by plasmodium [177]. This can be mostly due to the reality that the majority of plants do not generate extremely effective secondary metabolites, which are specific and can be used for antibacterial purposes because of having different defense systems [178]. However, because of the appearance as well as widespread antimicrobial-resistant genes amongst the microorganisms, plants are also exposed to anxiety that causes them to supply more effective antibiotics (capable against β-lactamase producing multidrug-resistant microorganisms) [179]. Consequently, it is progressively possible for one to find new antibiotics from plant derivatives and consider that such sample could also be composed of antimicrobial-contaminated sites. In keeping with Segal et al. (2006) [180], totally dissimilar histone proteins, which coil with a particular deoxyribonucleic acid sequence and make advanced nucleosomes that discourage binding of polymerases. Of that, simply a small fraction of these would come with assays to a degree wherever it is probable to hypothesize the type of activity of the secondary metabolites to permit direct evaluation of their cytotoxicity. Therefore, the shortage of data on the nature of activity primarily protects the substitution of antimicrobials by the treatment with plant secondary metabolites. To measure this appropriately, many approaches may be observed for explanation of the intense mechanism of secondary metabolites from plant origin, to be utilized as antibacterial agents. Individually, the foremost distinguished ways out have emerged which are accessible through the utilization of proteomics. Proteomic identification has been the main methodology that permits assessment of proteomic appearance after the treatment and management of clusters of cells. This may outline the type of activity by plant-determined antibiotics and the regulatory pathways concerned. Recognized proteomic strategies embody two-dimensional gel electrophoresis, stable isotopic labeling method, and elevated resolution, each one followed by recognition via mass spectrometry. Additionally, transcriptomic methodologies may also be utilized to evaluate proteomic information by gene expression profiling for a specific known protein. The quantitative RT-PCR is among the popular strategies performed to the repeated gene expression between the treated and un-treated control groups, revealing insight on the type of activity of the antibiotics derived from plant origin.

## 11. Conclusions

There have been reports of an expanding prevalence of ESBLs in various regions of the world. The high-risk patients are known to be the individuals who are infected with ESBL-producing strains that can render medicines insufficient on them. Thus, there is a critical requirement for immediately recognizable proof and proper policy directions to reduce the predominance of ESBLs. Urgent work is required to grow faster, cost-effective, and reliable analytic devices just as new effective therapies. Plant extracts and essential oil can create antimicrobial and antifungal properties. They effectively affect individuals from the fauna and rumen microflora that can be useful to profitability and health in the veterinary world. The progressing pattern which causes bacterial diseases is, at present, totally resistant to the entirety of current day traditional drugs already capable of destroying the contamination. Thus, the utilization of synergistic treatment doses or regimens joining purified compounds or plant extracts have been effective for the treatment of antimicrobial-resistant Gram-negative bacteria.

## Figures and Tables

**Table 1:** Classification of β-lactamases.

Ambler Class	Bush Group	Characteristics of β-Lactamases	Number of Enzymes
C	1	Often chromosomal enzymes in Gram-negative but some are plasmid coded. Not inhibited by clavulanic acid.	51
A	2a	Staphylococcal and enterococcal penicillinases.	23
	2b	Broad-spectrum β-lactamases including TEM-1 and SHV-1, mainly occurring in Gram-negative.	16
	2be	Extended-spectrum β-lactamases (ESBL).	200
	2br	Inhibitor-resistant TEM (IRT) β-lactamases.	24
	2c	Carbenicillin-hydrolyzing enzymes.	19
	2d	Cloxacillin (oxacillin)-hydrolyzing enzymes.	31
	2e	Cephalosporinases inhibited by clavulanic acid.	20
	2f	Carbapenem-hydrolyzing enzyme inhibited by clavulanic acid.	4
B	3	Metallo-enzymes that hydrolyze carbapenems and other β-lactams except monobactams. Not inhibited by clavulanic acid.	24
D	4	Miscellaneous enzymes that do not fit into other groups.	9

**Table 2:** Plants with antimicrobial activity.

Common Name	Scientific Name	Compound	Class	Activity	RELATIVE TOXICITY	References
Aloe	*Aloe barbadensis*, *Aloe vera*	Latex	Complex mixture	*Salmonella*	2.7	[73]
Apple	*Malus sylvestris*	Phloretin	Flavonoid derivative	General	3.0	[74]
Ashwagandha	*Withaniasomniferum*	Withafarin A	Lactone	Bacteria, fungi	0.0	
Basil	*Ocimum basilicum*	Essential oils	Terpenoids	*Salmonella*, bacteria	2.5	[75]
Black pepper	*Piper nigrum*	Piperine	Alkaloid	Fungi, *Lactobacillus*, *Micrococcus, E. coli*, *E. faecalis*	1.0	[76]
Blueberry	*Vaccinium* spp.	Fructose	Monosaccharide	*E. coli*		[77]
Coca	*Erythroxylum coca*	Cocaine	Alkaloid	Gram-negative and-positive cocci	0.5	
Green tea	*Camellia sinensis*	Catechin	Flavonoid	General *Shigella*	2.0	[78]
Turmeric	*Curcuma longa*	Curcumin	Terpenoids	Bacteria, protozoa		[79]
Potato	*Solanum* *tuberosum*	-		*Solanum tuberosum*	2.0	[80]
Onion	*Allium cepa*	Allicin	Sulfoxide	Bacteria, Candida		[81]
Goldenseal	*Hydrastis* *canadensis*	Berberine, hydrastine	Alkaloids	Bacteria, Giardia duodenale, trypanosomes	2.0	[82]

**Table 3:** Major classes of antimicrobial compounds from plants.

Class	Subclass	Example(s)	Mechanism	References
Phenolics	Simple phenols	Catechol	Substrate deprivation	[83]
		Epicatechin	Membrane disruption	[84]
	Phenolic acids	Cinnamic acid	Hydrogen atom transfer, sequential proton loss electron transfer.	[85]
	Quinones	Hypericin	Bind to adhesins, complex with cell wall, inactivate enzymes	[86]
	Flavonoids	Chrysin	Bind to adhesins	[87]
	Flavones		Complex with cell wall	
		Abyssinone	Inactivate enzymes Inhibit HIV reverse transcriptase	[88]
	Flavonols	Totarol	Control the accumulation of reactive oxygen species	[89]
	Tannins	Ellagitannin	Bind to proteins Bind to adhesins Enzyme inhibition Substrate deprivation Complex with cell wall Membrane disruption Metal ion complexation	[90]
	Coumarins	Warfarin	Interaction with eukaryotic DNA (antiviral activity)	[91]
Terpenoids, essential oils		Capsaicin	Membrane disruption	[92]
Alkaloids		Berberine	Intercalate into cell wall and/or DNA	[93]
Lectins and polypeptides		Mannose-specific agglutinin Fabatin	Block viral fusion or adsorption Form disulfide bridges	[94]
Polyacetylenes		8S-Heptadeca-2(*Z*),9(*Z*)-diene- 4,6-diyne-1,8-diol	Pleiotropic profile of bioactivity	[95]

**Table 4:** Antibacterial activity of tested plant extracts [99].

Plant Species		a	b	c	d	e	f	g	h	i
		MIC (mg/mL)								
***Cichoriumintybus***	E	5	10	10	10	20	20	20	5	2.5
	Et	2.18	2.18	2.18	8.75	8.75	2.18	2.18	2.18	1.09
	Ac	2.5	2.5	2.5	5	5	2.5	2.5	2.5	2.5
***Salvia officinalis***	E	5	5	5	>20	10	>20	>20	>20	2.5
	Et	10	20	20	>20	>20	>20	>20	>20	2.5
	Ac	0.03	0.15	0.31	20	1.25	20	0.31	0.156	0.019
***Clinopodium vulgare***	E	1.25	>20	>20	>20	20	>20	20	2.5	20
	Et	0.625	20	10	10	10	10	10	2.5	10
	Ac	1.25	20	10	10	10	20	10	0.625	10
***Cytisus nigricans***	E	2.5	20	20	10	10	20	20	5	1.25
	Et	5	20	20	20	20	20	20	5	5
	Ac	2.5	20	20	10	20	>20	20	2.5	10
***Dorycniumpentaphyllum***	E	5	10	20	10	5	20	20	2.5	10
	Et	1.25	20	20	10	10	>20	20	1.25	20
	Ac	1.25	20	20	5	5	20	10	1.25	10

a. *B. subtilis*; b. *K. pneumoniae*; c. *S. aureus*; d. *P. aeruginosa*; e. *P. mirabilis*; f. *E. coli*; g. *E. coli* ATCC 25922; h. *S. aureus* ATCC 25923; i. *P. aeruginosa* ATCC 27853; E: ethanol extract; Et: ethyl acetate extract; Ac: acetone extract.

**Table 5:** List of antibiotic resistance modifying plant extracts against different microorganisms.

Plant Scientific Name	Part Used	Microorganisms	Modulation of Resistance	Method of Study	References
*Rosmarinus officinalis*	Aerial part	*S. aureus*	MDR efflux inhibition	Ethidium bromide efflux assay	[139]
*Lycopus europaeus*	N/A	*S. aureus*	-	-	[140]
*Fissistigma cavaleriei*	Root	*P. aeruginosa*	β-lactamase inhibition	β-lactamase inhibitory assay	[141]
*Cardiospermum grandiflorum*	Leaves	*S. aureus*	-	-	[142]
*Momordica charantia* L.	Leaves	MRSA	Efflux pump inhibition	Efflux pump inhibitory assay	[143]
*Mentha arvensis* L.	Leaves	*E. coli*	-	-	[144]
*Turnera ulmifolia* L.	Leaves	MRSA	-	-	[145]
*Catha edulis*	Leaves	*Streptococcus oralis*, *Streptococcus sanguis*, *Fusobacterium* *nucleatum*	-	-	[146]
*Punica granatum*	Fruit	MRSA	Efflux pump inhibition	Time–kill assay, β-lactamase production detection, ethidium bromide efflux assay	[147]

**Table 6:** Cell targets of membrane-active compounds and its mode of study.

Targets	Mode of Study	Substances	References
Cell morphology: Alteration of cell shape or surface structure of the cell	Scanning electron micrograph (SEM)	*Cudrania tricuspidata* EO; *Allium sativum* EO;oregano EO; eugenol; epigallocatechin gallate	[170]
-	Transmission electron micrograph (TEM)	Tea tree oil; *Fortunella crassifolia* EO	[171]
Cytoplasmic membrane: Alteration of integrity and permeability	K+ leakage assay	Tea tree oil	[172]
-	Respiration assay	Tea tree oil	[172]
-	Propidium iodide uptake assay	Ferulic and gallic acids	[172]
Cell wall	OM permeability test	Ceratotoxin A; luteolin; flavonoids isolated from smaller galangal	[173]
-	Cell lysis assay	Oregano, thyme, clove EOs	[174]
Cell surface charge	Zeta potential measurement	Ferulic and gallic acids; lipids	[175]

## References

[1] Ma C., Zhu G., Li T., Zhao T. (2021). Chemical Composition, Antioxidant, Antimicrobial and Cholinesterase Inhibitory Activities of Essential Oils from the Leaves and Rhizomes of *Acorusmacrospadiceus* (Yamamoto) FN Wei et YK Li. J. Essent. Oil Bear..

[2] Shaikh S., Fatima J., Shakil S., Rizvi S.M.D., Kamal M.A. (2015). Antibiotic resistance and extended spectrum beta-lactamases: Types, epidemiology and treatment. Saudi J. Biol. Sci..

[3] Awari A., Nighute S., Khatoon M. (2013). Study of urinary isolates with reference to extended spectrum beta lactamases detection and antibiogram. J. Evol. Med. Dent. Sci..

[4] Larayetan R., Ololade Z.S., Ogunmola O.O., Ladokun A. (2019). Phytochemical constituents, antioxidant, cytotoxicity, antimicrobial, antitrypanosomal, and antimalarial potentials of the crude extracts of *Callistemon citrinus*. Evid. Based Complementary Altern. Med..

[5] World Health Organization (2014). Antimicrobial Resistance: Global Report on Surveillance.

[6] Dias D.A., Urban S., Roessner U. (2012). A historical overview of natural products in drug discovery. Metabolites.

[7] Fernandes P.A.D.S., Pereira R.L.S., Santos A.T.L.D., Coutinho H.D.M., Morais-Braga M.F.B., da Silva V.B., Costa A.R., Generino M.E.M., de Oliveira M.G., de Menezes S.A. (2022). Phytochemical Analysis, Antibacterial Activity and Modulating Effect of Essential Oil from *Syzygiumcumini* (L.) Skeels. Molecules.

[8] Bezerra J.W.A., Rodrigues F.C., Pereira da Cruz R., Silva L.E.D., do Amaral W., Andrade Rebelo R., Begnini I.M., Fonseca Bezerra C., Iriti M., Varoni E.M. (2020). Antibiotic potential and chemical composition of the essential oil of *Piper caldense* C. DC. (Piperaceae). Appl. Sci..

[9] Valli M., Pivatto M., Danuello A., Castro-Gamboa I., Silva D.H.S., Cavalheiro A.J., Araújo Â.R., Furlan M., Lopes M.N., Bolzani V.D.S. (2012). Tropical biodiversity: Has it been a potential source of secondary metabolites useful for medicinal chemistry?. Química Nova.

[10] Bezerra J.W.A., Costa A.R., de Freitas M.A., Rodrigues F.C., de Souza M.A., da Silva A.R.P., Dos Santos A.T.L., Linhares K.V., Coutinho H.D.M., de Lima Silva J.R. (2019). Chemical composition, antimicrobial, modulator and antioxidant activity of essential oil of *Dysphaniaambrosioides* (L.) Mosyakin&Clemants. Comp. Immunol. Microbiol. Infect. Dis..

[11] Sharifi-Rad J., Sureda A., Tenore G.C., Daglia M., Sharifi-Rad M., Valussi M., Tundis R., Sharifi-Rad M., Loizzo M.R., Ademiluyi A.O. (2017). Biological activities of essential oils: From plant chemoecology to traditional healing systems. Molecules.

[12] Fisher J.F., Mobashery S. (2010). Enzymology of Bacterial Resistance. Comprehensive Natural Products II Chemistry and Biology. Enzym. Enzym. Mech..

[13] Bonnet R. (2004). Growing group of extended-spectrum β-lactamases: The CTX-M enzymes. Antimicrob. Agents Chemother..

[14] Drawz S.M., Bonomo R.A. (2010). Three decades of β-lactamase inhibitors. Clin. Microbiol. Rev..

[15] Loder B., Newton G.G.F., Abraham E.P. (1961). The cephalosporin C nucleus (7-aminocephalosporanic acid) and some of its derivatives. Biochem. J..

[16] Kundu S., Chakravarty I., Ojha S., Kundu K. (2019). Design and Development of Antibiotic Fermentation Using Different Processing Strategies: Challenges and Perspectives. Appl. Microbiol. Bioeng. Interdiscip. Approach.

[17] Morin R.B., Jackson B.G., Flynn E.H., Roeske R.W., Andrews S.L. (1969). Chemistry of cephalosporin antibiotics. XIV. Reaction of cephalosporin C with nitrosyl chloride. J. Am. Chem. Soc..

[18] Goffin C., Ghuysen J.M. (1998). Multimodular penicillin-binding proteins: An enigmatic family of orthologs and paralogs. Microbiol. Mol. Biol. Rev..

[19] Poole K. (2004). Resistance to β-lactam antibiotics. Cell. Mol. Life Sci..

[20] Macheboeuf P., Di Guilmi A.M., Job V., Vernet T., Dideberg O., Dessen A. (2005). Active site restructuring regulates ligand recognition in class A penicillin-binding proteins. Proc. Natl. Acad. Sci. USA.

[21] Sacco E., Cortes M., Josseaume N., Rice L.B., Mainardi J.L., Arthur M. (2014). Serine/threonine protein phosphatase-mediated control of the peptidoglycan cross-linking L, D-transpeptidase pathway in *Enterococcus faecium*. mBio.

[22] Goswami M., Subramanian M., Kumar R., Jass J., Jawali N. (2016). Involvement of antibiotic efflux machinery in glutathione-mediated decreased ciprofloxacin activity in *Escherichia coli*. Antimicrob. Agents Chemother..

[23] Okamoto K., Gotoh N., Nishino T. (2002). Alterations of susceptibility of *Pseudomonas aeruginosa* by overproduction of multidrug efflux systems, MexAB-OprM, MexCD-OprJ, and MexXY/OprM to carbapenems: Substrate specificities of the efflux systems. J. Infect. Chemother..

[24] Costa S.S., Viveiros M., Rosato A.E., Melo-Cristino J., Couto I. (2015). Impact of efflux in the development of multidrug resistance phenotypes in *Staphylococcus aureus*. BMC Microbiol..

[25] Melano R., Corso A., Petroni A., Centrón D., Orman B., Pereyra A., Moreno N., Galas M. (2003). Multiple antibiotic-resistance mechanisms including a novel combination of extended-spectrum β-lactamases in a *Klebsiella pneumoniae* clinical strain isolated in Argentina. J. Antimicrob. Chemother..

[26] Vetting M.W., Magnet S., Nieves E., Roderick S.L., Blanchard J.S. (2004). A bacterial acetyltransferase capable of regioselective N-acetylation of antibiotics and histones. Chem. Biol..

[27] Schwarz S., Kehrenberg C., Doublet B., Cloeckaert A. (2004). Molecular basis of bacterial resistance to chloramphenicol and florfenicol. FEMS Microbiol. Rev..

[28] Nakamura A., Miyakozawa I., Nakazawa K., O’Hara K., Sawai T. (2000). Detection and characterization of a macrolide 2′-phosphotransferase from a *Pseudomonas aeruginosa* clinical isolate. Antimicrob. Agents Chemother..

[29] Matsuoka M., Sasaki T. (2004). Inactivation of macrolides by producers and pathogens. Curr. Drug Targets Infect Disord..

[30] Houang E.T., Chu Y.W., Lo W.S., Chu K.Y., Cheng A.F. (2003). Epidemiology of rifampin ADP-ribosyltransferase (arr-2) and metallo-β-lactamase (bla IMP-4) gene cassettes in class 1 integrons in *Acinetobacter* strains isolated from blood cultures in 1997 to 2000. Antimicrob. Agents Chemother..

[31] Benton B., Breukink E., Visscher I., Debabov D., Lunde C., Janc J., Mammen M., Humphrey P. (2007). O258 Telavancin inhibits peptidoglycan biosynthesis through preferential targeting of transglycosylation: Evidence for a multivalent interaction between telavancin and lipid II. Int. J. Antimicrob. Agents.

[32] Meziane-Cherif D., Stogios P.J., Evdokimova E., Egorova O., Savchenko A., Courvalin P. (2015). Structural and functional adaptation of vancomycin resistance VanT serine racemases. mBio.

[33] Arthur M., Molinas C., Bugg T.D., Wright G.D., Walsh C.T., Courvalin P. (1992). Evidence for in vivo incorporation of D-lactate into peptidoglycan precursors of vancomycin-resistant enterococci. Antimicrob. Agents Chemother..

[34] Bugg T.D., Wright G.D., Dutka-Malen S., Arthur M., Courvalin P., Walsh C.T. (1991). Molecular basis for vancomycin resistance in *Enterococcus faecium* BM4147: Biosynthesis of a depsipeptide peptidoglycan precursor by vancomycin resistance proteins VanH and VanA. Biochemistry.

[35] Park I.S., Lin C.H., Walsh C.T. (1996). Gain of D-alanyl-D-lactate or D-lactyl-D-alanine synthetase activities in three active-site mutants of the *Escherichia coli* D-alanyl-D-alanine ligase B. Biochemistry.

[36] Paterson D.L., Bonomo R.A. (2005). Extended-spectrum β-lactamases: A clinical update. Clinic. Microbiol. Rev..

[37] Bush K., Fisher J.F. (2011). Epidemiological expansion, structural studies, and clinical challenges of new β-lactamases from gram-negative bacteria. Annual Rev. Microbiol..

[38] Ambler R.P., Coulson A.F., Frère J.M., Ghuysen J.M., Joris B., Forsman M., Levesque R.C., Tiraby G., Waley S.G. (1991). A standard numbering scheme for the class A beta-lactamases. Biochem. J..

[39] Livermore D.M., Struelens M., Amorim J., Baquero F., Bille J., Canton R., Henning S., Gatermann S., Marchese A., Mittermayer H. (2002). Multicentre evaluation of the VITEK 2 Advanced Expert System for interpretive reading of antimicrobial resistance tests. J. Antimicrob. Chemother..

[40] Tzouvelekis L.S., Bonomo R.A. (1999). SHV-type beta-lactamases. Curr. Pharm. Des..

[41] Datta N., Kontomichalou P. (1965). Penicillinase synthesis controlled by infectious R factors in Enterobacteriaceae. Nature.

[42] Bois S.D., Marriott M.S., Amyes S.G.B. (1995). TEM-and SHV-derived extended-spectrum β-lactamases: Relationship between selection, structure and function. J. Antimicrob. Chemother..

[43] Gazouli M., Tzelepi E., Markogiannakis A., Legakis N.J., Tzouvelekis L.S. (1998). Two novel plasmid-mediated cefotaxime-hydrolyzing β-lactamases (CTX-M-5 and CTX-M-6) from *Salmonella typhimurium*. FEMS Microbiol. Lett..

[44] Tzouvelekis L.S., Tzelepi E., Tassios P.T., Legakis N.J. (2000). CTX-M-type β-lactamases: An emerging group of extended-spectrum enzymes. Int. J. Antimicrob. Agents.

[45] Bush K., Jacoby G.A. (2010). Updated functional classification of β-lactamases. Antimicrob. Agents Chemother..

[46] Humeniuk C., Arlet G., Gautier V., Grimont P., Labia R., Philippon A. (2002). β-Lactamases of *Kluyvera ascorbata*, probable progenitors of some plasmid-encoded CTX-M types. Antimicrob. Agents Chemother..

[47] Olson A.B., Silverman M., Boyd D.A., McGeer A., Willey B.M., Pong-Porter V., Daneman N., Mulvey M.R. (2005). Identification of a progenitor of the CTX-M-9 group of extended-spectrum β-lactamases from *Kluyvera georgiana* isolated in Guyana. Antimicrob. Agents Chemother..

[48] Bush K., Jacoby G.A., Medeiros A.A. (1995). A functional classification scheme for beta-lactamases and its correlation with molecular structure. Antimicrob. Agents Chemother..

[49] Weldhagen G.F., Poirel L., Nordmann P. (2003). Ambler class A extended-spectrum β-lactamases in Pseudomonas aeruginosa: Novel developments and clinical impact. Antimicrob. Agents Chemother..

[50] Poirel L., Le Thomas I., Naas T., Karim A., Nordmann P. (2000). Biochemical sequence analyses of GES-1, a novel class A extended-spectrum β-lactamase, and the class 1 integron In52 from *Klebsiella pneumoniae*. Antimicrob. Agents Chemother..

[51] Philippon L.N., Naas T., Bouthors A.T., Barakett V., Nordmann P. (1997). OXA-18, a class D clavulanic acid-inhibited extended-spectrum beta-lactamase from Pseudomonas aeruginosa. Antimicrob. Agents Chemother..

[52] Bauernfeind A., Stemplinger I., Jungwirth R., Ernst S., Casellas J.M. (1996). Sequences of beta-lactamase genes encoding CTX-M-1 (MEN-1) and CTX-M-2 and relationship of their amino acid sequences with those of other beta-lactamases. Antimicrobial Antimicrob. Agents Chemother..

[53] Neuhauser M.M., Weinstein R.A., Rydman R., Danziger L.H., Karam G., Quinn J.P. (2003). Antibiotic resistance among gram-negative bacilli in US intensive care units: Implications for fluoroquinolone use. JAMA.

[54] Vahaboglu H., Coskunkan F., Tansel O., Ozturk R., Sahin N., Koksal İ., Kocazeybek B., Tatman-Otkun M., Leblebicioglu H., Ozinel M.A. (2001). Clinical importance of extended-spectrum β-lactamase (PER-1-type)-producing *Acinetobacter* spp. and *Pseudomonas aeruginosa* strains. J. Med. Microb..

[55] Bradford P.A. (2001). Extended-spectrum β-lactamases in the 21st century: Characterization, epidemiology, and detection of this important resistance threat. Clin. Microb. Rev..

[56] Naas T., Oxacelay C., Nordmann P. (2007). Identification of CTX-M-type extended-spectrum-β-lactamase genes using real-time PCR and pyrosequencing. Antimicrob. Agents Chemother..

[57] Siegel J.D., Rhinehart E., Jackson M., Chiarello L. (2006). Management of multidrug-resistant organisms in healthcare settings. Am. J. Infect. Control.

[58] Kahlmeter G. (2008). Breakpoints for intravenously used cephalosporins in Enterobacteriaceae—EUCAST and CLSI breakpoints. Clin. Microbiol. Infect..

[59] Clinical and Laboratory Standards Institute (2002). Performance Standards for Antimicrobial Disk and Dilution Susceptibility Tests for Bacteria Isolated from Animals, Approved Standard.

[60] Health Protection Agency National Standard Methods, (15 July 2008). www.gov.uk/government/publications/health-protection-agency-annual-report-and-accounts-2008.

[61] Spanu T., Sanguinetti M., Tumbarello M., D’Inzeo T., Fiori B., Posteraro B., Santangelo R., Cauda R., Fadda G. (2006). Evaluation of the new VITEK 2 extended-spectrum beta-lactamase (ESBL) test for rapid detection of ESBL production in Enterobacteriaceae isolates. J. Clin. Microbiol..

[62] Wiegand I., Geiss H.K., Mack D., Sturenburg E., Seifert H. (2007). Detection of extended-spectrum beta-lactamases among Enterobacteriaceae by use of semiautomated microbiology systems and manual detection procedures. J. Clin. Microbiol..

[63] Pitout J.D.D., Hamilton N., Church D.L., Nordmann P., Poirel L. (2007). Development and clinical validation of a molecular diagnostic assay to detect CTX-M-type β-lactamases in Enterobacteriaceae. Clin. Microb. Infect..

[64] Batchelor M., Hopkins K., Threlfall E.J., Clifton-Hadley F.A., Stallwood A.D., Davies R.H., Liebana E. (2005). bla CTX-M genes in clinical Salmonella isolates recovered from humans in England and Wales from 1992 to 2003. Antimicrob. Agents Chemother..

[65] Woodford N., Fagan E.J., Ellington M.J. (2006). Multiplex PCR for rapid detection of genes encoding CTX-M extended-spectrum β-lactamases. J. Antimicrob. Chemother..

[66] Birkett C.I., Ludlam H.A., Woodford N., Brown D.F., Brown N.M., Roberts M.T., Milner N., Curran M.D. (2007). Real-time TaqMan PCR for rapid detection and typing of genes encoding CTX-M extended-spectrum β-lactamases. J. Med. Microbiol..

[67] Ensor V.M., Livermore D.M., Hawkey P.M. (2007). A novel reverse-line hybridization assay for identifying genotypes of CTX-M-type extended-spectrum β-lactamases. J. Antimicrob. Chemother..

[68] Geissman T.A. (1963). Flavonoid compounds, tannins, lignins and, related compounds. Comp. Biochem..

[69] Schultes R.E. (1978). The kingdom of plants. Med. Earth..

[70] Dejonghe W., Russinova E. (2017). Plant chemical genetics: From phenotype-based screens to synthetic biology. Plant Physiol..

[71] Gorlenko C.L., Kiselev H.Y., Budanova E.V., Zamyatnin A.A., Ikryannikova L.N. (2020). Plant secondary metabolites in the battle of drugs and drug-resistant bacteria: New heroes or worse clones of antibiotics?. Antibiotics.

[72] Forni C., Facchiano F., Bartoli M., Pieretti S., Facchiano A., D’Arcangelo D., Norelli S., Valle G., Nisini R., Beninati S. (2019). Beneficial role of phytochemicals on oxidative stress and age-related diseases. BioMed Res. Int..

[73] Martinez M.J., Betancourt J., Alonso-Gonzalez N., Jauregui A. (1996). Screening of some Cuban medicinal plants for antimicrobial activity. J. Ethnopharmacol..

[74] Hunter M.D., Hull L.A. (1993). Variation in concentrations of phloridzin and phloretin in apple foliage. Phytochemistry.

[75] Wan J., Wilcock A., Coventry M.J. (1998). The effect of essential oils of basil on the growth of *Aeromonas hydrophila* and *Pseudomonas fluorescens*. J. Appl. Microbiol..

[76] Ghoshal S., Prasad B.K., Lakshmi V. (1996). Antiamoebic activity of *Piper longum* fruits against *Entamoeba histolytica* in vitro and in vivo. J. Ethnopharmacol..

[77] Ofek I., Goldhar J., Sharon N. (1996). Anti-Escherichia coli adhesin activity of cranberry and blueberry juices. Towar. Anti-Adhes. Ther. Microb. Dis..

[78] Vijaya K., Ananthan S., Nalini R. (1995). Antibacterial effect of theaflavin, polyphenon 60 (*Camellia sinensis*) and *Euphorbia hirta* on *Shigella* spp.—A cell culture study. J. Ethnopharmacol..

[79] Apisariyakul A., Vanittanakom N., Buddhasukh D. (1995). Antifungal activity of turmeric oil extracted from *Curcuma longa* (Zingiberaceae). J. Ethnopharmacol..

[80] Hufford C.D., Jia Y., Croom E.M., Muhammed I., Okunade A.L., Clark A.M., Rogers R.D. (1993). Antimicrobial compounds from Petalostemum purpureum. J. Nat. Prod..

[81] Vohora S.B., Rizwan M., Khan J.A. (1973). Medicinal uses of common Indian vegetables. Planta Med..

[82] Freiburghaus F., Kaminsky R., Nkunya M.H.H., Brun R. (1996). Evaluation of African medicinal plants for their in vitro trypanocidal activity. J. Ethnopharmacol..

[83] Peres M.T., Delle Monache F., Cruz A.B., Pizzolatti M.G., Yunes R.A. (1997). Chemical composition and antimicrobial activity of *Croton urucurana* Baillon (Euphorbiaceae). J. Ethnopharmacol..

[84] Toda M., Okubo S., Ikigai H., Suzuki T., Suzuki Y., Hara Y., Shimamura T. (1992). The protective activity of tea catechins against experimental infection by *Vibrio cholerae* O1. Microbiol. Immunol..

[85] Fernandez M.A., Garcia M.D., Saenz M.T. (1996). Antibacterial activity of the phenolic acids fractions of *Scrophulariafrutescens* and *Scrophulariasambucifolia*. J. Ethnopharmacol..

[86] Duke J.A. (1985). CRC Handbook of Medicinal Herbs.

[87] Perrett S., Whitfield P.J., Sanderson L., Bartlett A. (1995). The plant molluscicide *Millettiathonningii* (Leguminosae) as a topical antischistosomal agent. J. Ethnopharmacol..

[88] Brinkworth R.I., Stoermer M.J., Fairlie D.P. (1992). Flavones are inhibitors of HIV-1 proteinase. Biochem. Biophy. Res. Commun..

[89] Kubo I., Muroi H., Himejima M. (1993). Combination effects of antifungal nagilactones against *Candida albicans* and two other fungi with phenylpropanoids. J. Nat. Prod..

[90] Haslam E. (1996). Natural polyphenols (vegetable tannins) as drugs: Possible modes of action. J. Nat. Prod..

[91] Keating G.J., O’kennedy R. (1997). The chemistry and occurrence of coumarins. Coumarins Biol. Appl. Mode Action.

[92] Cichewicz R.H., Thorpe P.A. (1996). The antimicrobial properties of chile peppers (Capsicum species) and their uses in Mayan medicine. J. Ethnopharmacol..

[93] Rahman A., Choudhary M.I. (1995). Diterpenoid and steroidal alkaloids. Nat. Prod. Rep..

[94] Meyer J.J.M., Afolayan A.J., Taylor M.B., Erasmus D. (1997). Antiviral activity of galangin isolated from the aerial parts of *Helichrysumaureonitens*. J. Ethnopharmacol..

[95] Estevez-Braun A., Estevez-Reyes R., Moujir L.M., Ravelo A.G., Gonzalez A.G. (1994). Antibiotic activity and absolute configuration of 8S-heptadeca-2 (Z), 9 (Z)-diene-4,6-diyne-1,8-diol from *Bupleurumsalicifolium*. J. Nat. Prod..

[96] Radulovic N.S., Blagojevic P.D., Stojanovic R.Z.Z., Stojanovic N.M. (2013). Antimicrobial plant metabolites: Structural diversity and mechanism of action. Curr. Med. Chem..

[97] Saleem M., Nazir M., Shaiq A.M., Hussain H., Lee Y.S., Riaz N., Jabbar A. (2010). Antimicrobial natural products: An update on future antibiotic drug candidates. Nat. Prod. Rep..

[98] Buzzini P., Arapitsas P., Goretti M., Branda E., Turchetti B., Pinelli P., Ieri F., Romani A. (2008). Antimicrobial and antiviral activity of hydrolysable tannins. Mini Rev. Med. Chem..

[99] Stefanović O.D. (2018). Synergistic Activity of Antibiotics and Bioactive Plant Extracts: A Study Against Gram-Positive and GramNegative Bacteria. Bact. Pathog. Antibact. Control..

[100] Chang S.T., Chen P.F., Chang S.C. (2001). Antibacterial activity of leaf essential oils and components from *Cinnamomumosmophloeum*. J. Ethnopharmacol..

[101] Imai H., Osawa K., Yasuda H., Hamashima H., Arai T., Sasatsu M. (2001). Inhibition by the essential oils of peppermint and spearmint of the growth of pathogenic bacteria. Microbios.

[102] Elgayyar M., Draughon F.A., Golden D.A., Mount J.R. (2001). Antimicrobial activity of essential oils from plants against selected pathogenic and saprophytic microorganisms. J. Food Protect..

[103] Ohno T., Kita M., Yamaoka Y., Imamura S., Yamamoto T., Mitsufuji S., Kodama T., Kashima K., Imanishi J. (2003). Antimicrobial activity of essential oils against *Helicobacter pylori*. Helicobacter.

[104] Langeveld W.T., Veldhuizen E.J., Burt S.A. (2014). Synergy between essential oil components and antibiotics: A review. Crit. Rev. Microbiol..

[105] Su C.H., Wang J.T., Hsiung C.A., Chien L.J., Chi C.L., Yu H.T. (2012). Increase of Carbapenem-Resistant *Acinetobacter baumannii* Infection in Acute Care Hospitals in Taiwan: Association with Hospital Antimicrobial Usage. PLoS ONE.

[106] Luqman S., Dwivedi G.R., Darokar M.P., Kalra A., Khanuja S.P. (2007). Potential of rosemary oil to be used in drug-resistant infections. Altern. Ther. Health Med..

[107] Hemaiswarya S., Kruthiventi A.K., Doble M. (2008). Synergism between natural products and antibiotics against infectious diseases. Phytomedicine.

[108] Prabuseenivasan S., Jayakumar M., Ignacimuthu S. (2006). In vitro antibacterial activity of some plant essential oils. BMC Complemen. Altern. Med..

[109] Van Vuuren S.F., Suliman S., Viljoen A.M. (2009). The antimicrobial activity of four commercial essential oils in combination with conventional antimicrobials. Lett. Appl. Microbiol..

[110] Krishnamurti C., Rao S.C. (2016). The isolation of morphine by Serturner. Indian J. Anaesth..

[111] Jeruto P., Nyangacha R.M., Mutai C. (2015). In vitro and in vivo antiplasmodial activity of extracts of selected Kenyan medicinal plants. Afr. J. Pharm. Pharmacol..

[112] McMahon J.B., Currens M.J., Gulakowski R.J., Buckheit R.W., Lackman-Smith C., Hallock Y.F., Boyd M.R. (1995). Michellamine B, a novel plant alkaloid, inhibits human immunodeficiency virus-induced cell killing by at least two distinct mechanisms. Antimicrob. Agents Chemother..

[113] McDevitt J.T., Schneider D.M., Katiyar S.K., Edlind T.D. (1996). Berberine: A candidate for the treatment of diarrhea in AIDS patients, abstr. 175. Proceedings of the Program and Abstracts of the 36th Interscience Conference on Antimicrobial Agents and Chemotherapy.

[114] Ohanu E.C., Inyang-Etoh P.C. (2015). The efficacy of plant extracts on cecalamebiasis in rats. Vet. Sci. Dev..

[115] Omulokoli E., Khan B., Chhabra S.C. (1997). Antiplasmodial activity of four Kenyan medicinal plants. J. Ethnopharmacol..

[116] Dahanukar S.A., Kulkarni R.A., Rege N.N. (2000). Pharmacology of Medicinal Plants and Natural Prod ucts. Indian J. Pharmacol..

[117] Barghash S.M. (2016). Evaluation of in vitro and in vivo activities of some medicinal plants against trypanosomiasis. Int. J. Adv. Res..

[118] Lee S.M., Kim M.S., Hayat F., Shin D. (2019). Recent advances in the discovery of novel antiprotozoal agents. Molecules.

[119] Tajner-Czopek A., Gertchen M., Rytel E., Kita A., Kucharska A.Z., Sokół-Łętowska A. (2020). Study of antioxidant activity of some medicinal plants having high content of caffeic acid derivatives. Antioxidants.

[120] Martinez M., Poirrier P., Chamy R., Prüfer D., Schulze-Gronover C., Jorquera L., Ruiz G. (2015). *Taraxacumofficinale* and related species—An ethnopharmacological review and its potential as a commercial medicinal plant. J. Ethnopharmacol..

[121] Gonçalves S., Romano A. (2019). Inhibitory properties of phenolic compounds against enzymes linked with human diseases. Phenolic Compounds-Biological Activity.

[122] Rasheed M.U., Thajuddin N., Ahamed P., Teklemariam Z., Jamil K. (2014). Antimicrobial drug resistance in strains of *Escherichia coli* isolated from food sources. Rev. Ins. Med. Trop. Pau..

[123] Pandit R., Awal B., Shrestha S.S., Joshi G., Rijal B.P., Parajuli N.P. (2020). Extended-spectrum β-lactamase (ESBL) genotypes among multidrug-resistant uropathogenic Escherichia coli clinical isolates from a teaching hospital of Nepal. Interdiscip. Perspect. Infect. Dis..

[124] Langdon A., Crook N., Dantas G. (2016). The effects of antibiotics on the microbiome throughout development and alternative approaches for therapeutic modulation. Genome Med..

[125] Petrovska B.B. (2012). Historical review of medicinal plants’ usage. Pharmacogn. Rev..

[126] Chaudhary H.S., Yadav J., Shrivastava A.R., Singh S., Singh A.K., Gopalan N. (2013). Antibacterial activity of actinomycetes isolated from different soil samples of Sheopur (A city of central India). J. Adv. Pharm. Tech. Res..

[127] Gilani S.A., Kikuchi A., Shinwari Z.K., Khattak Z.I., Watanabe K.N. (2007). Phytochemical, pharmacological and ethnobotanical studies of RhazyastrictaDecne. Phytother. Res. Int. J. Dev. Pharmacol. Toxicol. Eval. Nat. Prod. Deriv..

[128] Khan R., Islam B., Akram M., Shakil S., Ahmad A., Ali S.M., Siddiqui M., Khan A.U. (2009). Antimicrobial activity of five herbal extracts against multi drug resistant (MDR) strains of bacteria and fungus of clinical origin. Molecules.

[129] Chopra R.N. (1956). Glossary of Indian Medicinal Plants.

[130] Fleming A. Penicillin. Nobel Lecture. 11 December 1945. https://www.nobelprize.org/nobelprizes/medicine/laureates/1945/fleming-lecture.pdf.

[131] Rabe T., Van Staden J. (1997). Antibacterial activity of South African plants used for medicinal purposes. J. Ethnopharmacol..

[132] Agarwal S.S. (2005). Clinically Useful Herbal Drugs.

[133] Kamboj V.P. (2000). Herbal medicine. Curr. Sci..

[134] Burt S. (2004). Essential oils: Their antibacterial properties and potential applications in foods—A review. Int. J. Food Microbiol..

[135] Moore-Neibel K., Gerber C., Patel J., Friedman M., Ravishankar S. (2012). Antimicrobial activity of lemongrass oil against *Salmonella enterica* on organic leafy greens. J. Appl. Microbiol..

[136] Aleksic V., Knezevic P. (2014). Antimicrobial and antioxidative activity of extracts and essential oils of *Myrtuscommunis* L.. Microbiol. Res..

[137] Tyagi A.K., Malik A. (2012). Morphostructural damage in food-spoiling bacteria due to the lemon grass oil and its vapour: SEM, TEM, and AFM investigations. Evid. Based Complementary Altern. Med..

[138] Liu Y., Gallardo-Moreno A.M., Pinzon-Arango P.A., Reynolds Y., Rodriguez G., Camesano T.A. (2008). Cranberry changes the physicochemical surface properties of E. coli and adhesion with uroepithelial cells. Colloids Surf. B.

[139] Oluwatuyi M., Kaatz G.W., Gibbons S. (2004). Antibacterial and resistance modifying activity of Rosmarinusofficinalis. Phytochemistry.

[140] Gibbons S., Oluwatuyi M., Veitch N.C., Gray A.I. (2003). Bacterial resistance modifying agents from Lycopuseuropaeus. Phytochemistry.

[141] Yang Z., Niu Y., Le Y., Ma X., Qiao C. (2010). Beta-lactamase inhibitory component from the roots of Fissistigmacavaleriei. Phytomedicine.

[142] Petra O.N., Franklin C.K., Wilfred N.O. (2012). Cardiospermum grandiflorum leaf extract potentiates amoxicillin activity on Staphylococcus aureus. J. Med. Plants Res..

[143] Coutinho H.D., Costa J.G., Falcão-Silva V.S., Siqueira-Júnior J.P., Lima E.O. (2010). Effect of *Momordicacharantia* L. in the resistance to aminoglycosides in methicilin-resistant *Staphylococcus aureus*. Com. Immuno. Micro. Infec. Dis..

[144] Coutinho H.D., Costa J.G., Lima E.O., Falcão-Silva V.S., Siqueira-Júnior J.P. (2008). Enhancement of the antibiotic activity against a multiresistant *Escherichia coli* by *Mentha arvensis* L. and chlorpromazine. Chemotherapy.

[145] Coutinho H.D., Costa J.G., Lima E.O., Falcão-Silva V.S., SiqueiraJúnior J.P. (2009). Herbal therapy associated with antibiotic therapy: Potentiation of the antibiotic activity against methicillin–resistant Staphylococcus aureus by *Turneraulmifolia* L.. BMC Comp. Alt. Med..

[146] Al-hebshi N., Al-haroni M., Skaug N. (2006). In vitro antimicrobial and resistance-modifying activities of aqueous crude khat extracts against oral microorganisms. Arch. Oral Biol..

[147] Braga L.C., Leite A.A., Xavier K.G., Takahashi J.A., Bemquerer M.P., Chartone-Souza E., Nascimento A.M. (2005). Synergic interaction between pomegranate extract and antibiotics against Staphylococcus aureus. Can. J. Microbiol..

[148] Singh B.R., Singh V., Singh R.K., Ebibeni N. (2011). Antimicrobial activity of lemongrass (*Cymbopogon citratus*) oil against microbes of environmental, clinical and food origin. Int. Res. J. Pharm. Pharmacol..

[149] Singh B.R., Singh V., Ebibeni N., Singh R.K. (2013). Antimicrobial and herbal drug resistance in enteric bacteria isolated from faecal droppings of common house lizard/gecko (Hemidactylusfrenatus). Int. J. Microbiol..

[150] Brown J.C., Jiang X. (2008). Prevalence of antibiotic-resistant bacteria in herbal products. J. Food Prot..

[151] Ujam N.T., Oli A.N., Ikegbunam M.N., Adikwu M.U., Esimone C.O. (2013). Antimicrobial resistance evaluation of organisms isolated from liquid herbal products manufactured and marketed in South Eastern Nigeria. Br. J. Pharm. Res..

[152] Atanasov A.G., Zotchev S.B., Dirsch V.M. (2021). Natural products in drug discovery: Advances and opportunities. Nat. Rev. Drug Discov..

[153] Rafiq Z., Narasimhan S., Haridoss M., Vennila R., Vaidyanathan R. (2017). Punicagranatum rind extract: Antibiotic potentiator and efflux pump inhibitor of multidrug resistant *Klebsiella pneumoniae* clinical isolates. Asian J. Pharm. Clin. Res..

[154] Cristo J.S., Matias E.F., Figueredo F.G., Santos J.F., Pereira N.L., Junior J.G., Aquino P.E., Nogueira M.N., Ribeiro-Filho J., Cunha F.A. (2016). HPLC profile and antibiotic-modifying activity of *Azadirachtaindica* A. Juss (Meliaceae). Indus. Crops Prod..

[155] Inui T., Wang Y., Deng S., Smith D.C., Franzblau S.G., Pauli G.F. (2007). Counter-current chromatography based analysis of synergy in an anti-tuberculosis ethnobotanical. J. Chromatogr. A.

[156] Siriwong S., Teethaisong Y., Thumanu K., Dunkhunthod B., Eumkeb G. (2016). The synergy and mode of action of quercetin plus amoxicillin against amoxicillin-resistant Staphylococcus epidermidis. BMC Pharmacol. Toxicol..

[157] Phitaktim S., Chomnawang M., Sirichaiwetchakoon K., Dunkhunthod B., Hobbs G., Eumkeb G. (2016). Synergism and the mechanism of action of the combination of α-mangostin isolated from Garcinia mangostana L. and oxacillin against an oxacillin-resistant *Staphylococcus saprophyticus*. BMC Microbiol..

[158] Morita Y., Nakashima K.I., Nishino K., Kotani K., Tomida J., Inoue M., Kawamura Y. (2016). Berberine is a novel type efflux inhibitor which attenuates the MexXY-mediated aminoglycoside resistance in Pseudomonas aeruginosa. Front. Microbiol..

[159] Van Vuuren S., Viljoen A. (2011). Plant-based antimicrobial studies–methods and approaches to study the interaction between natural products. Planta Med..

[160] Neu H.C., Fu K.P. (1978). Clavulanic acid, a novel inhibitor of β-lactamases. Antimicrob. Agents Chemother..

[161] Musumeci R., Speciale A., Costanzo R., Annino A., Ragusa S., Rapisarda A., Pappalardo M.S., Iauk L. (2003). Berberis aetnensis C. Presl. extracts: Antimicrobial properties and interaction with ciprofloxacin. Int. J. Antimicrob. Agents.

[162] Nascimento G.G., Locatelli J., Freitas P.C., Silva G.L. (2000). Antibacterial activity of plant extracts and phytochemicals on antibiotic-resistant bacteria. Braz. J. Microbiol..

[163] Abreu A.C., McBain A.J., Simoes M. (2012). Plants as sources of new antimicrobials and resistance-modifying agents. Nat. Prod. Rep..

[164] Lorenzi V., Muselli A., Bernardini A.F., Berti L., Pagès J.M., Amaral L., Bolla J.M. (2009). Geraniol restores antibiotic activities against multidrug-resistant isolates from gram-negative species. Antimicrob. Agents Chemother..

[165] Yap P.S.X., Krishnan T., Yiap B.C., Hu C.P., Chan K.G., Lim S.H.E. (2014). Membrane disruption and anti-quorum sensing effects of synergistic interaction between L avandulaangustifolia (lavender oil) in combination with antibiotic against plasmid conferred multidrug resistant *Escherichia coli*. J. Appl. Microbiol..

[166] Yap P.S.X., Krishnan T., Chan K.G., Lim S.H.E. (2015). Antibacterial mode of action of *Cinnamomum verum* bark essential oil, alone and in combination with piperacillin, against a multi-drug-resistant Escherichia coli strain. J. Microbiol. Biotechnol..

[167] Maurya A., Dwivedi G.R., Darokar M.P., Srivastava S.K. (2013). Antibacterial and Synergy of Clavine Alkaloid Lysergol and its Derivatives Against Nalidixic Acid Resistant *Escherichia coli*. Chem. Biol. Drug Des..

[168] Stapleton P.D., Shah S., Anderson J.C., Hara Y., Hamilton-Miller J.M., Taylor P.W. (2004). Modulation of β-lactam resistance in Staphylococcus aureus by catechins and gallates. Int. J. Antimicrob. Agents.

[169] Oh E., Jeon B. (2015). Synergistic anti-*Campylobacter jejuni* activity of fluoroquinolone and macrolide antibiotics with phenolic compounds. Front. Microbiol..

[170] Bajpai V.K., Sharma A., Baek K.H. (2013). Antibacterial mode of action of Cudrania tricuspidata fruit essential oil, affecting membrane permeability and surface characteristics of food-borne pathogens. Food Control..

[171] Wang Y.W., Zeng W.C., Xu P.Y., Lan Y.J., Zhu R.X., Zhong K., Huang Y.N., Gao H. (2012). Chemical composition and antimicrobial activity of the essential oil of Kumquat (Fortunellacrassifolia Swingle) Peel. Int. J. Mol. Sci..

[172] Cox S.D., Mann C.M., Markham J.L., Bell H.C., Gustafson J.E., Warmington J.R., Wyllie S.G. (2000). The mode of antimicrobialaction of the essential oil of Melaleuca alternifolia (tea tree oil). J. Appl. Microbiol..

[173] Eumkeb G., Siriwong S., Phitaktim S., Rojtinnakorn N., Sakdarat S. (2012). Synergistic activity and mode of action of flavonoids isolated fromsmaller galangal and amoxicillin combinations against amoxicillin-resistant Escherichia coli. J. Appl. Microbiol..

[174] Jassim S.A., Naji M.A. (2003). Novel antiviral agents: A medicinal plant perspective. J. Appl. Microbiol..

[175] Borges A., Ferreira C., Saavedra M.J., Simoes M. (2013). Antibacterialactivity and mode of action of ferulic and gallic acids againstpathogenic bacteria. Microb. Drug Resist..

[176] Li D., Zhang S., Hao X., Ma K., Tan X., Wang Z., Li N. (1980). Pharmacologic study of colchicine-amide. Chin. Med. J..

[177] Miller L.H., Su X. (2011). Artemisinin: Discovery from the Chinese herbal garden. Cell.

[178] Dixon R.A. (2001). Natural products and plant disease resistance. Nature.

[179] Lewis K., Ausubel F.M. (2006). Prospects for plant-derived antibacterials. Nat. Biotech..

[180] Segal E., Fondufe-Mittendorf Y., Chen L., Thåström A., Field Y., Moore I.K., Wang J.P.Z., Widom J. (2006). A genomic code for nucleosome positioning. Nature.

